# Recovering wastewater RNA for virome sequencing by systematically optimized tangential-flow ultrafiltration and Nanotrap microbiome particles

**DOI:** 10.1128/aem.00777-25

**Published:** 2025-08-29

**Authors:** Emily Segelhurst, Jonathan E. Bard, Sydney Gallo, Vicky Huang, Alyssa Pohlman, Donald A. Yergeau, Jennifer A. Surtees, Ian M. Bradley, Yinyin Ye

**Affiliations:** 1 Department of Civil, Structural and Environmental Engineering, University at Buffalo 12292 , Buffalo, New York, USA; 2 UB Genomics and Bioinformatics Core, University at Buffalo 12292 , Buffalo, New York, USA; 3 Genetics, Genomics and Bioinformatics Graduate Program, Jacobs School of Medicine and Biomedical Sciences, University at Buffalo 12292 , Buffalo, New York, USA; 4 Department of Biochemistry, Jacobs School of Medicine and Biomedical Sciences, University at Buffalo 12292 , Buffalo, New York, USA; 5 Department of Microbiology and Immunology, Jacobs School of Medicine and Biomedical Sciences, University at Buffalo 12292 , Buffalo, New York, USA; 6 Research and Education in Energy, Environmental and Water (RENEW) Institute, University at Buffalo 12292 , Buffalo, New York, USA; Centers for Disease Control and Prevention, Atlanta, Georgia, USA

**Keywords:** genomic sequencing, whole-transcriptome shotgun sequencing, amplicon sequencing, crossflow filtration, virus concentration, wastewater-based epidemiology

## Abstract

Municipal wastewater harbors diverse RNA viruses, which are responsible for many emerging and reemerging diseases in humans, animals, and plants. Although genomic sequencing can be a high-throughput approach for profiling the RNA virome in wastewater, wastewater processing methods often influence sequencing outcomes. Here, we systematically evaluated two wastewater processing methods, tangential-flow ultrafiltration (TFF) and Nanotrap Microbiome A Particles, for detecting the target RNA virus severe acute respiratory syndrome coronavirus 2 (SARS-CoV-2) via amplicon sequencing and characterizing the RNA virome using whole-transcriptome shotgun sequencing. Our results from paired comparison tests showed that the TFF and Nanotrap methods recovered similar SARS-CoV-2 variants at the lineage level (analysis of similarity [ANOSIM] *R* = −0.012, *P* = 0.874). Optimizing automated procedures for the Nanotrap method and concentration factors for the TFF method was critical for achieving high-depth and high-breadth coverage of the target virus genome. Notably, the two methods enriched distinct RNA viromes from the same wastewater samples (ANOSIM *R* = 0.260, *P* = 0.002), with TFF samples showing 22-fold and 7-fold higher relative abundances of *Reoviridae* and *Coronaviridae*, respectively. These differences are likely due to the distinct virus concentration mechanisms employed by each method, which are influenced by liquid-solid partitioning of virus particles and interactions of viral surface proteins with ligands. Our findings underscore the importance of optimizing wastewater processing methods for genomic monitoring and have implications for broader environmental applications.

**IMPORTANCE:**

Wastewater genomic sequencing is an emerging technology for tracking viral infections within communities. However, different methods for concentrating viruses and extracting nucleic acids can influence the recoveries of RNA virome from wastewater. An in-depth understanding of virus concentration mechanisms and their impact on sequencing data quality and bioinformatic output would be critical to guide method selection and optimization. Specifically, this study systematically evaluated tangential-flow ultrafiltration and Nanotrap microbiome particles for their application to sequence SARS-CoV-2 and whole RNA virome from wastewater. Both methods yielded high-quality sequencing data for amplicon sequencing of SARS-CoV-2, but their outcomes diverged in the recovered RNA virome. We identified RNA viruses that are preferentially recovered by each of these two methods and proposed considerations of method selection for future studies of wastewater RNA virome.

## INTRODUCTION

Monitoring viral genomic sequences in wastewater is an effective tool for tracking infectious diseases within communities (1–3). Wastewater contains RNA viruses, many of which are responsible for emerging and reemerging diseases in humans, animals, and plants, including coronavirus, influenza virus, reovirus, and pepper mild mottle virus (4). These viruses can be shed in human or animal waste at high concentrations, eventually entering wastewater systems.

The feasibility of detecting viral RNA in wastewater relies on RNA stability. Although RNA viruses have a broad range of capacities to retain their infectivity in wastewater (5–7), their genetic materials inside the virions are relatively stable, depending on the wastewater matrices. For example, the *T*_90_ values (i.e., the time required to cause 90% decay of the original amount) of encapsidated viral RNA were as long as 8–12 days in untreated wastewater at around room temperature (8, 9). Without the protection of viral capsids, naked viral RNA degraded by 90% within a few minutes in raw wastewater (10). It is therefore practical to sequence RNA associated with virus particles in wastewater.

Many wastewater processing methods with various concentration mechanisms have been optimized and compared for their efficiencies of recovering severe acute respiratory syndrome coronavirus 2 (SARS-CoV-2) and other virus particles from wastewater, including liquid-solid phase partitioning (11), chemical aggregation (i.e., polyethylene glycol, aluminum or ferric salts, and polymers) (12, 13), ultracentrifugal forces (i.e., direct ultracentrifugation and cushion-based ultracentrifugation) (14), size exclusion (i.e., dead-end and tangential-flow filtration) (15), adsorption and desorption (i.e., U.S. Environmental Protection Agency standard method and charged membrane filtration) (16, 17), and protein-ligand affinity-based methods (i.e., Nanotrap microbiome particles) (18). Among those methods, wastewater samples concentrated by Nanotrap particles and ultrafiltration typically resulted in high success rates of recovering complete or near-complete SARS-CoV-2 RNA genomes from wastewater through amplicon-based whole-genome sequencing (19–21). The Nanotrap particles capture viruses through the interactions between the virus proteins and the affinity baits fixed on the magnetic bead surfaces (22, 23). In addition to SARS-CoV-2, the Nanotrap microbiome particles have been used to isolate and detect other microbes and viruses from a small volume of wastewater (~10 mL), such as norovirus, pepper mild mottle virus, and influenza virus (18, 24–26). In comparison, the ultrafiltration method first removes wastewater solids and debris through centrifugation and subsequently concentrates viruses in the clear supernatant using ultrafilter membranes. Previous studies of Nanotrap and ultrafiltration methods primarily focused on comparing their ability to detect human viruses using PCR-based assays or hybridization capture-based sequencing (27). Their enrichment of RNA virome when applied with amplicon-based sequencing and whole-transcriptome shotgun sequencing is less investigated.

In this study, we aimed to systematically evaluate Nanotrap and tangential-flow ultrafiltration (TFF) methods for recovering wastewater viruses and to develop an insightful understanding of the impact of wastewater processing methods on sequencing the target RNA virus or whole RNA virome. Given that Nanotrap particles rely on protein-ligand interactions, which might not perform equally well for all RNA viruses, we hypothesized that TFF would capture a more diverse RNA virome than Nanotrap. To investigate this, we employed two sequencing approaches. First, amplicon-based sequencing was used to compare the breadth and depth coverage of SARS-CoV-2 genome, diversity at the lineage level, and variant composition from the same wastewater samples. Second, whole-transcriptome shotgun sequencing was conducted to compare the diversity of RNA virome recovered by both methods at the family level. Our findings inform practical recommendations for the selection and optimization of wastewater processing methods for future wastewater RNA virome analysis.

## RESULTS

### Breadth and depth coverage of SARS-CoV-2 genome by Nanotrap and TFF

**Fig 1:**
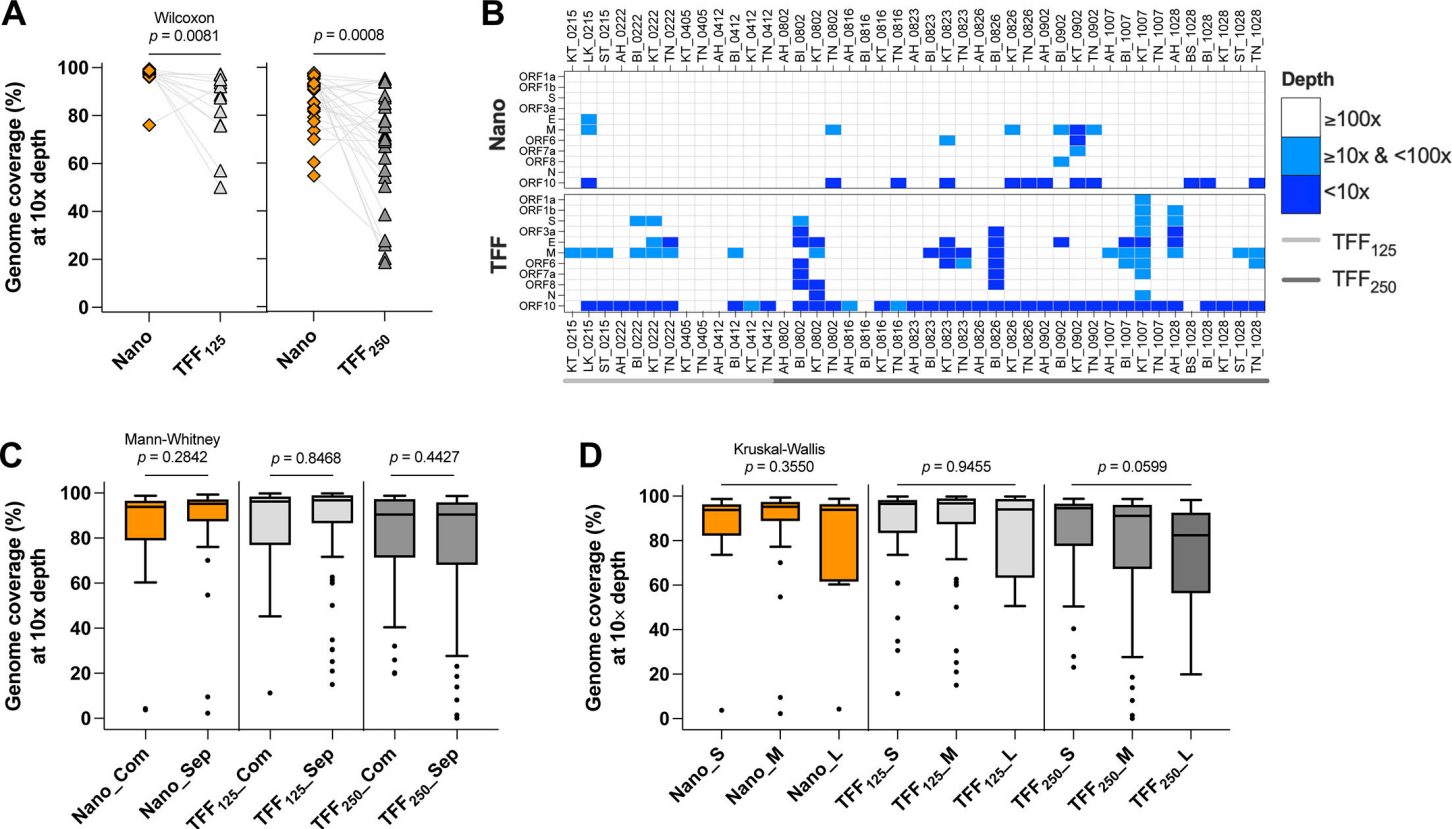
Breadth and depth coverage of SARS-CoV-2 genome from wastewater influent samples by Nanotrap microbiome particles (Nano), tangential-flow ultrafiltration processing 125 mL wastewater (TFF_125_), and tangential-flow ultrafiltration processing 250 mL wastewater (TFF_250_). (**A**) Paired comparison of genome breadth coverage by Nanotrap and TFF_125_ (*n* = 13) and by Nanotrap and TFF_250_ (*n* = 30). The gray lines connect the same wastewater samples. (**B**) Paired comparison of sequencing depth at the gene level by Nanotrap and TFF. (**C**) Comparison of genome coverages between the combined (Com) and separate (Sep) sewer systems. (**D**) Comparison of genome coverages among small- (S, <10 million gallons per day [MGD]), medium- (M, 10–100 MGD), and large-scale (L, >100 MGD) wastewater treatment facilities.

Two batches of paired comparisons were conducted to evaluate the breadth coverage of SARS-CoV-2 genome at 10× depth (hereafter referred to as coverage) when the wastewater was processed by different methods: Nanotrap and TFF_125_ in the first batch (*n* = 13) and Nanotrap and TFF_250_ in the second batch (*n* = 30). As a result, TFF_125_ yielded significantly lower median coverage of the SARS-CoV-2 genome (88.6%, interquartile range [IQR] = 76.0%–93.9%) than Nanotrap (97.8%, IQR = 97.1%–98.7%; Wilcoxon matched-pair signed-rank test, *P* = 0.0081; Fig. 1A). The processing of a larger volume of wastewater by TFF_250_ led to a significantly greater variation in the median genome coverage (71.2%, IQR = 58.4%–86.8%) relative to Nanotrap (91.7%, IQR = 81.7%–94.8%; Wilcoxon matched-pair signed-rank test, *P* = 0.0008; Fig. 1A). Furthermore, the Nanotrap method showed high sequencing depth across the SARS-CoV-2 genome (>100×), including the S gene (Fig. 1B), whereas the TFF method led to a greater proportion of genes sequenced at low depth (<10×), particularly the M and ORF10 genes (Fig. 1B). These findings suggest that the Nanotrap method has a more consistent performance in achieving high depth coverage of the SARS-CoV-2 genome from wastewater samples than the TFF method.

The paired comparison was conducted with a limited number of wastewater samples. To test whether the quality of sequencing data by Nanotrap, TFF_125_, and TFF_250_ was biased by wastewater matrices, we used unpaired, longitudinal sequencing data to evaluate the effects of sewer types and treatment plant scales on each processing method. For all three methods, there were no statistical differences in the median genome coverages between the combined and separate sewer systems (all Mann-Whitney tests *P* > 0.05, Fig. 1C). In addition, the genome coverages by each of the three methods were not significantly different among the small (S, <10 million gallons per day [MGD]), medium (M, 10–100 MGD), and large (L, >100 MGD) scales of wastewater treatment facilities (all Kruskal-Wallis tests *P* > 0.05, Fig. 1D). Although a clear trend of decreasing median genome coverage with increasing wastewater treatment scales was observed in TFF_250_ samples, the differences were statistically insignificant (post hoc Dunn’s multiple comparison test, all *P*_adj_ > 0.05; Table S1). These results indicate that the selection of sewersheds and sampling period are unlikely to influence the results of our paired comparison tests. Moreover, the Nanotrap and TFF methods are both robust wastewater processing methods in combination with downstream sequencing techniques.

### Detection of SARS-CoV-2 lineages and variants by Nanotrap and TFF

**Fig 2:**
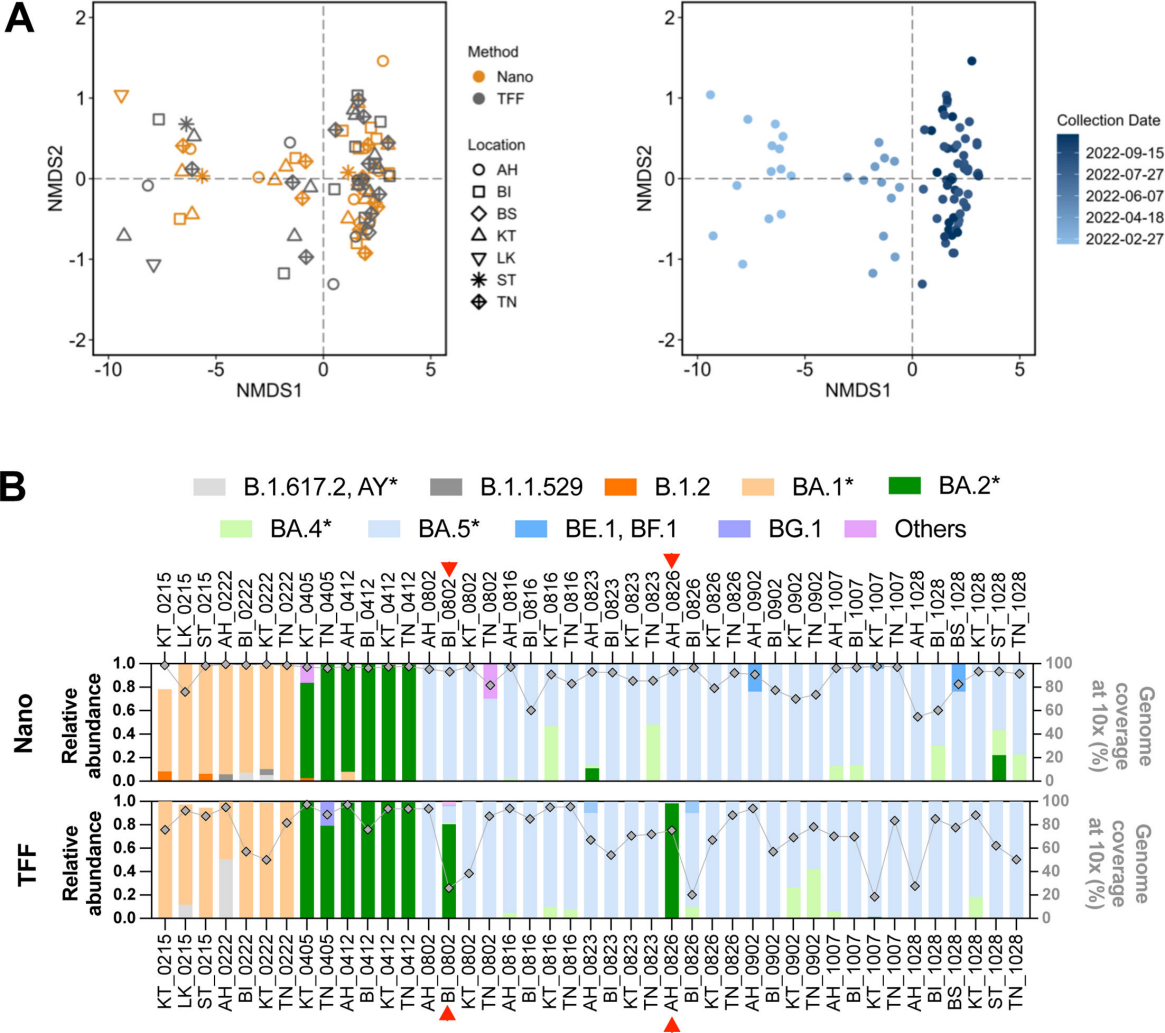
Comparison of SARS-CoV-2 lineage and variant composition detected from wastewater samples processed by Nanotrap microbiome particles (Nano) and tangential-flow ultrafiltration (TFF). (**A**) Non-metric multidimensional scaling (NMDS) plot of SARS-CoV-2 lineages by processing methods/locations and by collection dates. (**B**) Side-by-side comparison of Nanotrap and TFF for variant composition and genome coverage at 10× depth.

SARS-CoV-2 lineages detected from the same wastewater samples processed by Nanotrap and TFF were not significantly different in their richness, Shannon, and Simpson diversities within the samples (all Wilcoxon matched-pair signed-rank tests *P* > 0.05, Fig. S3). The collection data, rather than the wastewater processing method, was the primary driver of the separation of SARS-CoV-2 lineages, as shown in the non-metric multidimensional scaling (NMDS) plot (analysis of similarity [ANOSIM] *R* = 0.440, *P* = 0.001 for dates; ANOSIM *R* = −0.012, *P* = 0.874 for methods; Fig. 2A). This suggests that both methods were sensitive in identifying the emergence of new SARS-CoV-2 lineages over time.

To compare the relative abundance of SARS-CoV-2 variants by the two methods, we grouped lineages into 10 variants according to the surges of variants of concern and variants of interest during the periods of sampling seasons in Erie County, NY. The variant groups included AY* (B.1.617.2 and its sublineages), B.1.1.529, B.1.2, and BA.1* (BA.1 and its sublineages), BA.2* (BA.2 and its sublineages), BA.4* (BA.4 and its sublineages), BA.5* (BA.5 and its sublineages), BE.1 and BF.1, BG.1, and others. The Nanotrap and TFF methods detected very similar compositions of variants in most samples (Fig. 2B), except for BI_0802 and AH_0826 (Fig. 2B). In both samples, the Nanotrap method was biased toward BA.5*, while the TFF method favored detection of BA.2*. In the BI_0802 sample, Nanotrap resulted in 99.8% BA.5* (44.9% BA.5.1 and 54.9% BA.5.2), whereas the TFF processing yielded 80.5% BA.2* and 14.2% BA.5*. Notably, the TFF method covered only 25.9% of the SARS-CoV-2 genome from BI_0802, and the detected mutations were shared among multiple sublineages of BA.2* and BA.5* with each sublineage assigned the same relative abundance of 1.3% (Table S2); this low coverage reduces confidence in the lineage calling. In the AH_0826 sample, Nanotrap resulted in 99.8% BA.5* (69.9% BA.5.1, 13.6% BA.5.2, and 16.3% BA.5.2.1), whereas TFF yielded 98.3% BA.2* (98.2% BA.2.15 and 0.1% BA.2.18) (Table S2). This discrepancy is probably due to the low coverage of a specific region in the S gene by TFF (22,000–23,500 bases; Fig. S4), from which the corresponding amino acid mutations of L452R, F486V, and Q493R could distinguish BA.5.1/BA.5.2 from BA.2.15 (Fig. S5). Our results suggest that the failure to detect variant-specific mutations likely led to inaccurate estimates of variant relative abundance in wastewater samples.

### RNA virus families recovered by Nanotrap and TFF

We next evaluated the detection of whole RNA viruses from wastewater collected over 8 months from four local sewersheds (*n* = 12) and processed by Nanotrap and TFF. For the TFF method, we concentrated 250 mL of wastewater to yield high amounts of nucleic acids for sequencing (Fig. S6). We classified each of the shotgun sequencing reads against the pre-built expanded reference sequence database of Standard PlusPFP. This database contains archaeal, viral, bacterial, fungal, protozoan, plasmid, human, and plant reference genomes, allowing us to link RNA sequences to potential source organisms in wastewater. The Nanotrap method yielded 29.3%–55.6% unclassified reads, whereas the TFF method showed 13.9%–40.9% unclassified reads (Table S3).

**Fig 3:**
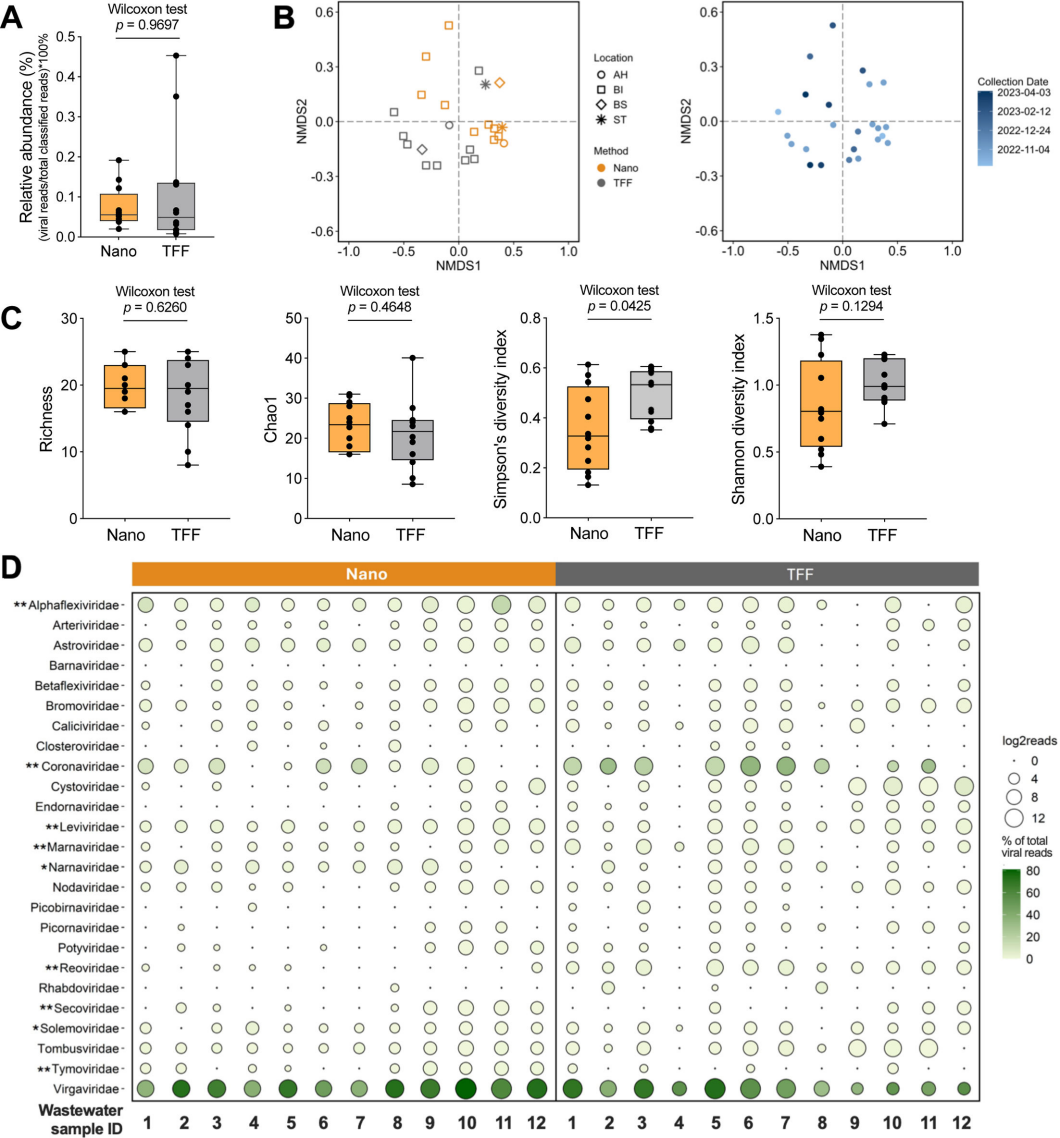
Diversity of RNA virus families recovered by Nanotrap microbiome particles (Nano) and tangential-flow ultrafiltration (TFF). (**A**) Relative read abundance of viruses (viral reads normalized to the total classified reads) in the Nano and TFF samples; (**B**) non-metric multidimensional scaling (NMDS) plot of RNA virus families; (**C**) alpha-diversity indices of richness, Chao1, Simpson’s, and Shannon’s indices; and (**D**) bubble plot of reads and fractions in total viral reads (%) for selected RNA virus families. For each virus family, median fractions were compared between Nanotrap and TFF using Wilcoxon matched-pair signed-rank tests. ** in front of the virus family name indicates *P* < 0.01; * indicates 0.01 ≤ *P* < 0.05.

In the total classified reads, the relative read abundance of viruses was low, ranging from 0.01% to 0.45% in both methods (Fig. 3A; Table S3). Although the TFF samples showed a slightly higher relative read abundance of viruses compared to the Nanotrap samples, the differences were not statistically significant (Wilcoxon matched-pair signed-rank tests, *P* = 0.9697). The low viral abundance allowed us to analyze the viral composition at the family level but did not allow a full phylogenetic analysis. At the family level, the wastewater RNA virome was clustered by the wastewater processing methods (ANOSIM *R* = 0.260, *P* = 0.002; Fig. 3B), rather than the collection dates (ANOSIM *R* = 0.005, *P* = 0.399; Fig. 3B). Alpha-diversity indices of richness, Chao1, and Shannon were not significantly different between these two methods (Wilcoxon matched-pair signed-rank tests, all *P* > 0.05; Fig. 3C). In contrast, Simpson’s diversity index, which emphasizes dominant RNA virus families, was significantly higher in TFF than in Nanotrap (Wilcoxon matched-pair signed-rank test, *P* = 0.0425; Fig. 3C). The mixed results from alpha-diversity indices suggest that Nanotrap and TFF recovered similar richness of RNA virus families, but they were different in the dominant RNA virus families (28).

To compare the dominant RNA virus families detected by Nanotrap and TFF, we filtered the data with a minimum of 10 unique reads in at least one sample by each method (27). Twenty-six RNA virus families were detected, and their fractions were calculated by normalizing the reads to the total classified viral reads (Table S4). We then compared the relative read proportion of these virus families in 12 wastewater samples prepared by Nanotrap and TFF. Nearly half of the classified viral reads in each of the Nanotrap and TFF samples were mapped to the virus families that infect plants and fungi as their major hosts, including *Bromoviridae*, *Narnaviridae*, *Secoviridae*, *Tombusviridae*, *Tymoviridae*, and *Virgaviridae* (Fig. 3D). Tobamoviruses, such as cucumber green mottle mosaic viruses and pepper mild mottle viruses in the family of *Virgaviridae*, are the dominant RNA viruses in municipal wastewater globally (29–33). RNA virus families with potential human and animal pathogens, including *Arteriviridae*, *Astroviridae*, *Caliciviridae*, *Coronaviridae*, *Picornaviridae*, and *Reoviridae* (*Sedoreoviridae*), consisted of approximately 4% and 22% of the total viral reads in the Nanotrap and TFF samples, respectively (Fig. 3D). In terms of RNA bacteriophages, both methods recovered small median fractions of *Cystoviridae* and *Leviviridae* (<1% of the total viral reads, Fig. 3D). Notably, TFF yielded significantly higher median read fractions of *Reoviridae* (22×), *Coronaviridae* (7×), and *Marnaviridae* (3×) than Nanotrap (Wilcoxon matched-pair signed-rank tests, *P* < 0.05; Fig. 3D; Table S5), while the Nanotrap enriched significantly higher fractions of *Tymoviridae* (141×), *Secoviridae* (20×), *Narnaviridae* (20×), *Leviviridae* (3×), *Aphaflexiviridae* (2×), and *Solemoviridae* (1.4×) than TFF (Wilcoxon matched-pair signed-rank tests, *P* < 0.05; Fig. 3D; Table S5). Collectively, our results demonstrate distinct enrichment patterns of RNA viromes recovered by the Nanotrap and TFF methods.

### Taxonomic diversity of non-viral RNA sequences recovered by Nanotrap and TFF

**Fig 4:**
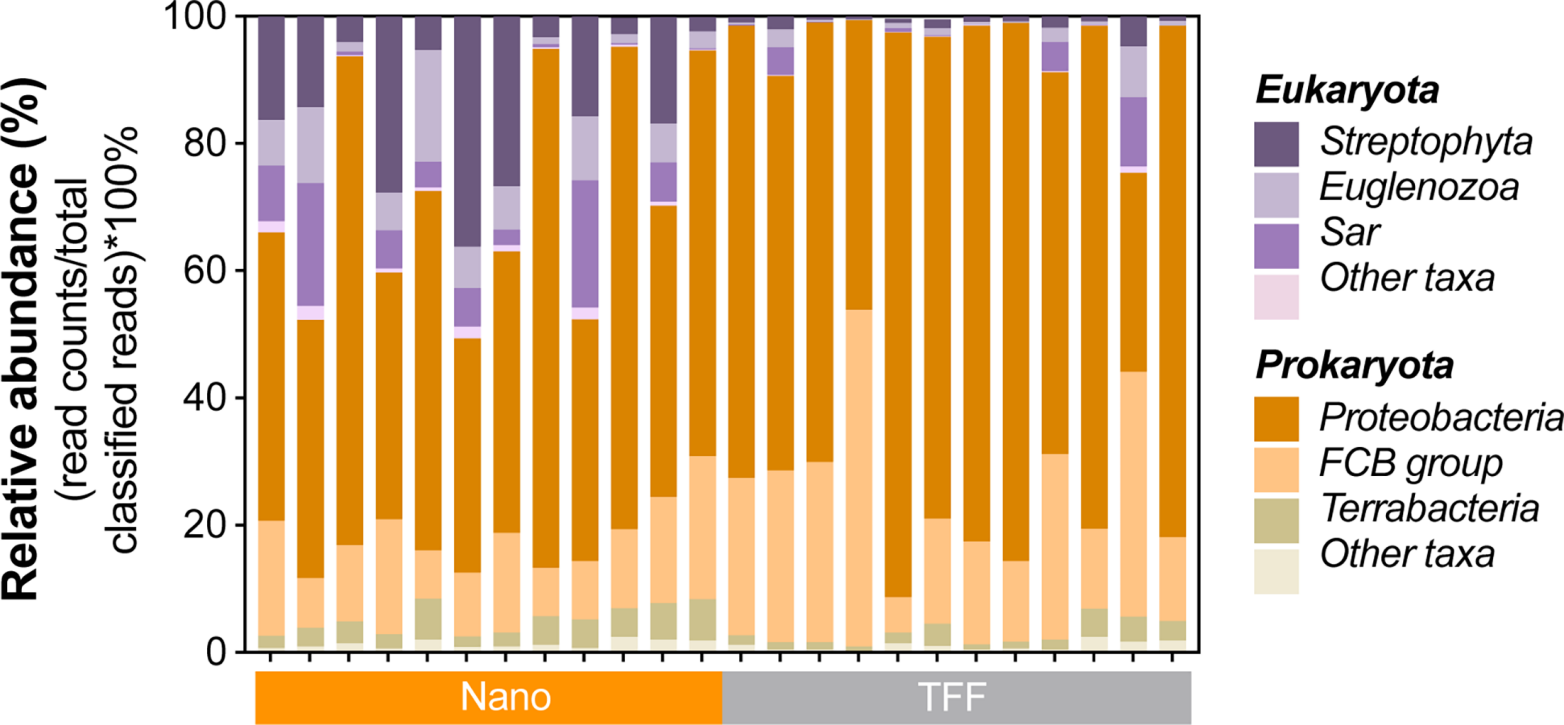
Relative read abundance of eukaryotic and eukaryotic phyla from the wastewater samples processed by Nanotrap microbiome particles (Nano) and tangential-flow ultrafiltration.

Although Nanotrap and TFF were applied to recover virus particles, greater than 99% of the total classified reads in the TFF and Nanotrap samples were eukaryotic and prokaryotic sequences (Fig. 4). The phyla of eukaryotes were dominant by Streptophyta, Euglenozoa, and Sar, and the prokaryotes were mainly Proteobacteria, FCB group, and Terrabacteria in our wastewater samples. Nanotrap and TFF resulted in different enrichments of prokaryotic and eukaryotic sequences. For example, the median relative read abundance of eukaryotes was significantly lower in the TFF samples than in the Nanotrap samples (Wilcoxon matched-pair signed-rank test, *P* = 0.0005; Fig. 4). This was not surprising because large eukaryotic cells were likely removed by the initial centrifugation step (10,000 × *g*, 15 min) in the TFF method. Many bacterial pathogens or opportunistic pathogens were detected, including the genera of *Acinetobacter*, *Aeromonas*, *Bacteroides*, *Clostridium* (*Clostridioides*), *Klebsiella*, *Pseudomonas*, *Staphylococcus*, and *Streptococcus* (Table S6). It is possible to apply either Nanotrap or TFF to monitor a broad range of prokaryotic and/or eukaryotic pathogens along with viruses for future wastewater-based epidemiology, but further validation is needed to compare wastewater signals with clinical cases.

## DISCUSSION

Characterizing genetic sequences of viruses in wastewater is valuable for tracking infectious diseases through wastewater-based epidemiology. Although a variety of wastewater processing methods and workflows have been applied for concentrating target viruses from wastewater matrices, we know little about their impact on RNA virome analysis. We conducted an apple-to-apple comparison of Nanotrap and TFF methods for isolating nucleic acids from wastewater samples collected over a long-term period across multiple wastewater treatment plants. Our results showed that, contrary to our hypothesis, the protein affinity-based Nanotrap method was not more selective for SARS-CoV-2 variants than the size exclusion-based TFF method. However, several RNA virus families varied in relative abundance between the two methods.

### Comparable detection of SARS-CoV-2 lineages and variant compositions from wastewater by Nanotrap and TFF

Despite different concentration mechanisms, the Nanotrap and TFF methods detected similar composition of SARS-CoV-2 variants from the same wastewater samples. This is probably because these two methods were applied in this study following optimal procedures for SARS-CoV-2 recovery. The genome coverage by the TFF method was consistent with the genome coverage reported for other ultrafiltration systems in either dead-end mode (i.e., Amicon or Centricon centrifugal filters) (34) or tangential mode (i.e., InnovaPrep system) (35). The Nanotrap method had greater variability in SARS-CoV-2 genome coverages. Manual extraction of virus particles from wastewater using Nanotrap yielded significantly lower genome coverage (1.5%– 30.0%) (24, 36) compared to automated procedures (>90%) (19). In our study, we applied a two-stage automated protocol including the virus enrichment from wastewater using the Nanotrap beads and then the nucleic acid extraction using microbiome particle-based extraction kits in the KingFisher system (Text S1 and S2). The automated steps are likely to facilitate thorough mixing of bead-wastewater and efficient collection of beads, thereby improving the recovery of SARS-CoV-2 nucleic acids from wastewater.

The explanation for the higher genome coverage by Nanotrap relative to TFF could be the presence of various amounts of sequencing inhibitors in the final nucleic acid extracts. As calculated from the volume of wastewater samples processed and the volume of nucleic acid finally extracted, the TFF_250_, TFF_125_, and Nanotrap methods concentrated wastewater by 4,167×, 2,083×, and 200×, respectively. Wastewater samples were highly concentrated by the TFF. Moreover, when the nucleic acids extracted from the TFF concentrates were diluted, higher concentrations of SARS-CoV-2 genes were measured, suggesting the presence of substances that inhibit the reverse transcription and/or amplification step (Fig. S7). Inhibition is not uncommon in wastewater. Microbial enzymes and chemical compounds discharged from industrial facilities can interfere with reverse transcription and oligo hybridization (37, 38). Yet the sources of inhibitory substances concentrated by the TFF system in this study are unclear. They were unlikely to be free metals or small chemicals because compounds with small molecular weights are readily filtered through the 30 kDa membrane, although any inhibitors might also be in complex with other larger molecules. It could also be due to the binding of ARCTIC primers to other environmental nucleic acids. More optimization efforts are needed to address the concentration of sequencing inhibitors when a large volume of wastewater is processed.

### Distinct selectivity of RNA virus families from wastewater by Nanotrap and TFF

Nanotrap and TFF preferentially enriched different RNA virus families from wastewater. This is probably related to different mechanisms of virus concentrations. Previous studies of Nanotrap particles showed that chemical compounds fixed on the bead surfaces such as Reactive Red 120, acrylic acid, vinyl sulfonic acid, and Cibacron blue F3GA (22, 23) had slightly reduced adsorption capacity of glycoproteins at pH 7–8 (39). The pH of our wastewater samples (~7.2) may affect the affinity interactions between the viral glycoproteins and the reactive dyes on the Nanotrap beads, possibly resulting in lower relative abundance of glycoprotein-containing rotaviruses and coronaviruses in the Nanotrap samples (40, 41) than in the TFF samples. One other possible explanation for the different virus recoveries between the two methods is virus partitioning to wastewater solids (42). The TFF preferentially recovers viruses in the liquid fraction of wastewater. Furthermore, different nucleic acid extraction kits used in the Nanotrap and TFF methods might influence viral communities (43). However, the MagMAX kits applied with the Nanotrap method effectively extracted RNA from rotaviruses and coronaviruses in stool samples (44, 45). Therefore, the nucleic acid extraction step is unlikely responsible for the low sequence reads of *Reoviridae* and *Coronaviridae* in Nanotrap samples. To gain further insights into virus recoveries using these two workflows, future studies incorporating spike-in control viruses containing glycosylated proteins or solid-partitioning features would be beneficial.

### Challenges in removing non-viral RNA sequences by current Nanotrap and TFF workflows

Compared to previous wastewater metagenomic sequencing studies, we found that the depletion of prokaryotic or eukaryotic reads through the wastewater concentration workflows alone is challenging. The classified bacterial reads were 20–50 times higher than the viral reads in the wastewater samples when concentrated by adsorption-elution (46), polyethylene glycol precipitation (46), and flocculation by acidic skim milk solution (32). Combining the metagenomic sequencing with probe-based techniques effectively enriches viral reads and enhances the sequencing sensitivity for the target virus panel (2, 3, 47), but the probe enrichment would miss emerging sequences. There are several possible explanations for the presence of high eukaryotic and prokaryotic reads in Nanotrap and TFF samples. Prokaryotic or eukaryotic cells are lysed in wastewater due to environmental stress or centrifugation (48). Moreover, microbes produce nano-sized extracellular vesicles that contain their genetic materials (49). The extracellular vesicles have dimensions and structures close to virus particles and thus cannot be separated by the size exclusion-based ultrafiltration (50). To improve the sensitivity of shotgun sequencing of wastewater virome, it is critical for future studies to investigate the sources of eukaryotic and prokaryotic reads and guide innovations in wastewater processing methods that indeed concentrate on viral genetics.

### Considerations of selecting appropriate wastewater processing methods for future wastewater virome studies

The selection of a wastewater processing method is important for RNA virome studies. We evaluated Nanotrap, based on protein-ligand affinity, and TFF, relying on size exclusion, side-by-side. Although both methods can recover similar communities of SARS-CoV-2 and their mutants when optimized, virus selectivity was observed when studying the whole RNA virome. The protein-ligand interactions rely on binding kinetics and binding capacity of ligands (51). Consequently, viruses with specific protein properties may not be highly enriched. To ensure reproducible recoveries, thorough mixing of samples (proteins) and beads (ligands) is necessary. We therefore recommend using automated steps instead of manual procedures when applying protein-ligand affinity-based methods.

Although TFF is based on size-exclusion mechanisms, which theoretically should concentrate all nano-sized viruses, the solid-removal step selects viruses in the liquid fraction for TFF concentration. Compared to the protein-ligand affinity method, the size-exclusion approach requires more time and energy input to recover viruses from wastewater. Additional investment in ultrafilter membranes needs to be considered for large-scale applications. Furthermore, wastewater contains substances that inhibit sequencing, making it crucial to optimize concentration factors to balance virus recovery with potential co-concentration of sequencing inhibitors.

## MATERIALS AND METHODS

### Sampling locations and wastewater sample collection

Influent wastewater samples were collected from seven local sewersheds (Table 1) between 3 September 2021 and 6 April 2023. Samples were collected as 24 h flow-weighted or time-weighted composites in high-density polyethylene sampling bottles. Sample bottles were pre-treated with 10% bleach for at least 30 min and then rinsed with MilliQ water. After collection, samples were transported to the laboratory at the University at Buffalo with ice packs and stored at 4 ℃ no more than 4 h prior to processing.

**TABLE 1:** Wastewater treatment facilities studied^*a*^

Sewershed	Permit flow (MGD)	Estimated population (2018 Census)	Rural, suburban, or urban	Sewer type
Tonawanda	2.5–5.0	14,873	Suburban	Combined
Lackawanna	4.5	17,859	Suburban/rural	Separate
Big sisters	7.7	29,853	Rural	Separate
South Town	16.0	94,616	Suburban/rural	Separate
Kenmore-Tonawanda	25.0	70,470	Suburban	Separate
Amherst	48.0	140,324	Suburban	Separate
Bird Island	180.0	437,357	Urban/rural	Combined

^*a*^ MGD, million gallons per day.

### Wastewater processing methods

Wastewater samples were spiked with bovine coronavirus (Zoetis, New Jersey) at a final concentration of approximately 10^7^ gene copies/L as a control. The samples were then aliquoted and concentrated using the TFF and Nanotrap methods as described below. The collection timeline for samples processed by Nanotrap, TFF_125_ (concentrating 125 mL wastewater with TFF), and TFF_250_ (processing 250 mL wastewater with TFF) is illustrated in Fig. S1. Nucleic acids were extracted from the concentrates using commercial RNA extraction kits, stored at −80°C, and submitted monthly to the UB Genomics and Bioinformatics Core for sequencing.

#### TFF

The TFF method followed the procedures optimized in a previous study with slight modifications (20). In brief, wastewater samples (125 or 250 mL) were first centrifuged at 10,000 × *g* for 15 min at 4°C. The cleared supernatant was then collected and fed through the TFF system using the Vivaflow 50R Crossflow system (Sartorius) with a 30 kDa 50R Hydrostart membrane (Sartorius) with a permeate flow rate of approxiamtely 8 mL/min. The TFF concentrate (25–30 mL) was immediately centrifuged over 5 mL of 20% (wt/vol) sucrose at 100,000 × *g* for 45 min at 4°C in an ultracentrifuge Optima XE-100 (Beckman Coulter). The pellets were resuspended in 200 µL of phosphate-buffered saline (Gibco, pH 7.4), from which the RNA was extracted using QIAamp viral RNA mini kits (Qiagen) and eluted in 60 µL of RNase-free water with sodium azide (AVE) buffer (Qiagen) following the manufacturer’s protocol. To clean the ultrafilter membranes, the TFF system was washed with 0.5 M sodium hydroxide solution warmed at 55°C. Our control experiments that concentrated autoclaved MilliQ water with the same procedures immediately after the membrane wash and then applied RT-qPCR assays to quantify the SARS-CoV-2 N2 gene and pepper mild mottle virus RNA (52, 53) suggested that the clean cycle effectively removed the SARS-CoV-2 RNA to the level below the limit of detection and removed >99.9% of pepper mild mottle virus RNA. We also tracked SARS-CoV-2 genome coverages over repeated use of the ultrafilter membranes and applied no more than 31 times of reuse per membrane (Fig. S2).

#### Nanotrap microbiome particles

Wastewater samples (9.6 mL) and autoclaved MilliQ water (9.6 mL negative controls) were evenly split into 4.8 mL and loaded into two separate KingFisher 24 deep-well plates (Sample Plate 1 and Sample Plate 2) at the matched position. Each 4.8 mL aliquot (wastewater samples or negative controls) in the deep-well plates was mixed with 50 µL Nanotrap Enhancement Reagent 1 (Ceres Nanosciences) and 75 µL Nanotrap Microbiome A Particles (Ceres Nanosciences). Virus particles from wastewater samples were enriched automatically following the wastewater virus capture protocol in the KingFisher Apex Purification System (Thermo Fisher Scientific) (Text S1). The extracted virus particles were finally eluted in 500 µL of MagMAX Microbiome Lysis Solution. Four hundred microliters of the lysates was then transferred to a 96 deep-well plate and mixed with 530 µL of MagMAX Binding Solution, 10 µL of MagMAX Proteinase K, and 20 µL of MagMAX DNA/RNA Binding Beads. The nucleic acids were washed in MagMAX Wash Buffer and then 80% ethanol, finally eluted in 50 µL of MagMAX Elution Buffer following the nucleic acid extraction protocol in the KingFisher Apex Purification System (Text S2).

### Amplicon sequencing of SARS-CoV-2 genome

SARS-CoV-2 genomes were sequenced following a modified ARTIC protocol in our previous study (20). Specifically, 8 µL of wastewater nucleic acid extracts were reverse transcribed to cDNA using LunaScript RT SuperMix Kit (New England Biolabs). The cDNA sample was then amplified with two primer pools in the ARTIC (v.4.0) nCOV-2019 amplicon panel (IDT) using Q5 Hot Start High-Fidelity 2× Master Mix (New England Biolabs). The amplicons from both pools were combined, cleaned with 1× AMPure XP beads (Beckman Coulter), and eluted in Qiagen EB buffer (10 mM Tris-HCl, pH 8.5) for library preparation. PhiX sequences (Illumina) were added to the pooled libraries at a concentration of 1% as an internal control. Sequencing was then performed on an Illumina NextSeq 500 (v.2.5) to obtain 75 bp paired-end reads.

### RNA library preparation and whole-transcriptome shotgun sequencing

Wastewater nucleic acid extracts were treated with DNase I for 10 min at 37°C, and capillary electrophoresis was performed on an Agilent 5200 Fragment Analyzer System. RNA concentrations were quantified using a Qubit Fluorometer with RNA Broad Range Assay kits (Invitrogen). RNA libraries were prepared with 100 ng of RNA per sample using Illumina Stranded Total RNA Prep Ligation with Ribo-Zero Plus Kit per the manufacturer’s protocol, which included steps to remove prokaryotic and eukaryotic ribosomal RNA and globin RNA. Following the completion of RNA library preparation, samples were rechecked for concentration and quality, and then libraries were pooled. The sequencer loading concentration of the pool library was determined using the sparQ Universal Library Quant Kit (QuantaBio). The pooled library (250 pM) was loaded onto a NovaSeq 6000 for sequencing to obtain 100 bp paired-end reads.

### Bioinformatics

For SARS-CoV-2 amplicon sequencing, the sequencing output files were analyzed with a bioinformatic pipeline developed in our previous study (20), including FASTQ file conversion, initial quality control, adapter trimming, and SARS-CoV-2 reference genome (MN908947.3) alignment. BCFtools (v.1.10.2) was then applied to generate a VCF file with a minimum depth per nucleotide position of 10× (54). The resulting VCF and depth files were used as input into Freyja (v.1.3.6) (https://github.com/andersen-lab/Freyja) to perform lineage composition analysis of known lineages (19). The Fryja algorithm was selected because complete genome coverage of SARS-CoV-2 is not necessarily required for lineage detection; additionally, Freyja uses a curated barcode library, allowing it to distinguish true lineage signals from random or artifactual variants.

The obtained whole-transcriptome shotgun sequencing data were first evaluated for quality using FastQC. Adaptors were then trimmed, and the resulting data were used as input into the Kraken2 algorithm (55) against the Standard PlusPFP database. This pre-built index contains sequences available in RefSeq for viral, archaea, plasmid, protozoa, fungi, and plants. Briefly, each sequence is broken into kmers and compared against a collection of RefSeq reference sequences to identify best-hit classification. The resulting hits were viewed in the Pavian browser (56) and extracted for statistical and biodiversity analyses.

### Statistical and biodiversity analyses

Wilcoxon matched-pair signed-rank tests were applied to assess statistical differences in median SARS-CoV-2 genome coverages from the same wastewater sample but prepared by two different processing methods. The medians of SARS-CoV-2 genome coverage were compared because the distinctions of SARS-CoV-2 genome coverage were non-normal. Mann-Whitney tests were used to compare statistical differences in median genome coverages between the combined and separate sewer systems. Kruskal-Wallis tests were employed to compare statistical differences in the median genome coverages among the small-, medium-, and large-scale sewersheds.

The relative abundance of SARS-CoV-2 lineages reported from Freyja and the relative read abundance of RNA virus families that normalized to the total classified reads were used for community diversity analysis. Alpha-diversity indices (evenness, Chao 1, Simpson’s, and Shannon) of SARS-CoV-2 lineages and RNA virus families were calculated in R (v.4.2.21) using package vegan (v.2.6.4) (https://github.com/vegandevs/vegan). The statistical comparisons of the alpha-diversity indices of viral communities recovered by Nanotrap and TFF were tested using Wilcoxon matched-pair signed-rank tests due to the non-normal distributions of the index values. The NMDS ordination of the Bray-Curtis distance followed by ANOSIM was applied to compare the beta diversity among different viromes. For all the statistical comparisons mentioned above, the null hypothesis was rejected when the *P* value was less than 0.05.

## Data Availability

The raw sequencing data required to reproduce the above findings are available to download from the National Center for Biotechnology Information Sequence Read Archive under the BioProject ID PRJNA1245320.

## SUPPLEMENTAL MATERIAL

Supplemental material aem.00777-25-s0001.docx Text S1, Fig. S1 to S7, and legends for Tables S1 to S6.

Supplemental tables aem.00777-25-s0002.xlsx Tables S1 to S6.

ASM does not own the copyrights to Supplemental Material that may be linked to, or accessed through, an article. The authors have granted ASM a non-exclusive, world-wide license to publish the Supplemental Material files. Please contact the corresponding author directly for reuse.
