# Supplementary material for: Recovering wastewater RNA for virome sequencing by systematically optimized tangential-flow ultrafiltration and Nanotrap microbiome particles
Source: Appl Environ Microbiol. 2025 Aug 29;91(10):e00777-25. doi: 10.1128/aem.00777-25 (PMC12542684; doi:10.1128/aem.00777-25)
Supplement: Supplemental material — Text S1, Fig. S1 to S7, and legends for Tables S1 to S6. [file aem.00777-25-s0001.docx]

Supplementary Materials

**Sequencing wastewater RNA virome using systematically optimized tangential-flow ultrafiltration and Nanotrap® microbiome particles**

Emily Segelhurst,^1^ Jonathan E. Bard,^2,3,5^ Sydney Gallo,^1^ Vicky Huang,^1^ Alyssa Pohlman,^2^ Donald A. Yergeau,^2^ Jennifer A. Surtees,^3,4,5^ Ian M. Bradley,^1,6^ Yinyin Ye^1,*^

^1^Department of Civil, Structural and Environmental Engineering, University at Buffalo, Buffalo, New York 14260, United States

^2^UB Genomics and Bioinformatics Core, University at Buffalo, Buffalo, New York 14203, United States

^3^Department of Biochemistry, Jacobs School of Medicine and Biomedical Sciences, University at Buffalo, Buffalo, New York 14203, United States

^4^Genetics, Genomics and Bioinformatics Graduate Program, Jacobs School of Medicine and Biomedical Sciences, University at Buffalo, Buffalo, New York 14203, United States

^5^Department of Microbiology and Immunology, Jacobs School of Medicine and Biomedical Sciences, University at Buffalo, Buffalo, New York 14203, United States

^6^Research and Education in Energy, Environmental and Water (RENEW) Institute, University at Buffalo, Buffalo, New York 14260, United States

_____________________________________

*Corresponding author: [yinyinye@buffalo.edu](mailto:yinyinye@buffalo.edu), Tel. (716) 645-4002

**Text S1.** KingFisher Apex Purification System-wastewater virus capture protocol using Nanotrap® microbiome particles

Wastewater samples (9.6 mL) were split equally and loaded in two KingFisher^TM^ 24 deep-well plates at the matched positions mixed with 50 µL Nanotrap® Enhancement Reagent 1 (Ceres Nanosciences) and 75 µL Nanotrap® Magnetic Virus Particles (now named as Nanotrap® Microbiome A Particles, Ceres Nanosciences). MilliQ water samples (9.6 mL) were used as negative controls and loaded in the same manner. A new KingFisher^TM^ 24 deep-well plate was loaded with 500 µL of MagMAX Microbiome Lysis Solution (ThermoFisher). The KingFisher^TM^ 24 deep-well tip comb was placed in one of the sample plates, and the virus extraction was run in the KingFisher™ Apex Purification System (ThermoFisher Scientific) following the steps below:

Initiation: Step type: Pick Up Tip; Tip type: 24 DW tip comb; Step 1 Bind Sample Plate 1: Step type: Bind; Precollect beads: Off; Release beads: On, 00:00:00; Heating & Cooling: Off; Mixing: #1 00:00:15, Medium, Looping 10; #2 00:00:45, Paused, Tip position above well; Postmix: On, 00:00:15, Medium; Collect beads: On, 5 Count 3 Seconds; Step 2 Drop Beads into Sample Plate 2: Step type: Mix; Precollect bead: Off; Release beads: On, 00:00:00; Heating & Cooling: Off; Mixing: #1 00:00:10 Bottom mix, Looping 1; #2 00:00:10, Medium; Postmix: off; Collect beads: off; Step 3 Collect Remainder from Sample 1: Step type: Collect Beads; Collect beads: Count 5, Collect time (s): 1; Step 4 Bind Sample 2; Step type: Bind; Precollect beads: Off; Release beads: On, 00:00:00; Heating & Cooling: Off; Mixing: #1 00:00:15 Medium, Looping 10; #2 00:00:45, Paused, Tip position above well; Postmix: On, 00:00:15, Medium; Collect beads: On, 5 Count, 3 Seconds; Step 5 Drop Beads into Elution: Step type: Mix; Precollect beads: Off; Release beads: On 00:00:00; Heating & Cooling: Off; Mixing: #1 00:00:10 Bottom mix, Looping 1; #2 00:00:10, Meidum; Postmix: Off; Collect beads: Off; Step 6 Collect Remainder from Sample 2: Step type: Collect Beads: Collect beads: Count 5 Collect time (s): 1; Step 7 Elute: Step type: Elute; Precollect beads: Off; Release beads: On, 00:00:10 Bottom mix; Heating & Colling: Off; Mixing: #1 00:00:15 Medium, Looping: 5; #2: 00:01:45, Paused, Tip position above well; Postmix: On, 00:00:15 Medium; Collect beads: On, 5 Count 5 Seconds; Step 8 Didcard Beads into Sample 2: Step type: Mix; Precollect beads: Off; Release beads: On 00:00:00; Heating & Cooling: Off; Mixing: #1 00:00:10, Bottom mix, Looping 1; #2 00:00:10, Medium; Postmix: Off; Collect beads: Off; Step 9 Collect Remainder from Elution: Step type: Collect Beads: Collect beads: Count 3, Collect time (s): 1; Final step: Step type: Leave Tip.

**Text S2.** KingFisher Apex Purification System – nucleic acid extraction protocol using MagMAX Viral/Pathogen Nucleic Acid Isolation Kit

From the previous step, 400 µL of lysates from each wastewater sample were loaded into a new KingFisher^TM^ 96 deep-well plate and mixed with 530 µL of MagMAX Binding Solution, 10 µL of MagMAX Proteinase K, and 20 µL of MagMAX DNA/RNA Binding Beads. Two new KingFisher^TM^ 96 deep-well plates were loaded with 1 mL of MagMAX Wash Buffer as the first wash solution and 1 mL of 80% ethanol as the second wash solution, respectively. A new KingFisher^TM^ 96 plate (not deep-well) was loaded with 50 µL of MagMAX Elution Buffer. The KingFisher^TM^ 96 deep-well tip comb was placed in the plate containing the lysate mixtures, and run the following steps for nucleic acid purification in the KingFisher™ Apex Purification System:

Initiation: Step type: Pick Up Tip; Tip type: 96 DW tip comb; Cycle 1: Step type: Lysis & Bind; Precollect beads: Off; Release beads: On 00:00:00; Heating & Cooling: On, 65 ºC, preheat On; Mixing: #1 00:05:00 Fast; Postmix: Off; Collect beads: On, 5 Count 0 Seconds; Cycle 2: Step type: Wash; Precollect beads: Off; Release beads: On, 00:00:20 Bottom mix; Heating & Cooling: Off; Mixing #1 00:00:10, Bottom mix, Looping 3; #2 00:00:10, Fast; Postmix: Off; Collect beads: On, 4 Count 1 Seconds; Cycle 3: Step type: Wash: Precollect beads: Off; Release beads: On, 00:00:20 Fast; Heating & Cooling: Off; Mixing #1 00:00:10, Bottom mix, Looping 2; #2 00:00:10, Fast; Postmix: Off; Collect beads: On, 3 Count, 1 Seconds; Cycle 4: Step type: Dry; Duration: 00:02:00, Outside well; Cycle 5: Step type: Elute; Precollect beads: Off; Release beads: On, 00:00:00; Heating & Cooling: On, 70 ºC, preheat On; Mixing: #1 00:00:15, Bottom mix, Looping 6; #2 00:00:45, Medium; Postmix: Off; Collect beads: On, 5 Count 0 Seconds; Cycle 5: Step type: Mix; Precollect beads: Off; Release beads: Off; Heating & Cooling: Off; Mixing: #1 00:01:30, Slow; Postmix: Off; Collect beads: Off; Final step: Step type: Leave Tip

**Figure S1**. Time distribution of wastewater samples used for pair comparison of SARS-CoV-2 amplicon sequencing, pair comparison of whole-transcriptome shotgun sequencing (RNA-Seq), and matrix effect assessment. Wastewater samples were concentrated by Nanotrap® microbiome particles (Nanotrap) and tangential-flow ultrafiltration (TFF) methods. The TFF was operated to concentrate 125 mL (TFF_125_) and 250 mL (TFF_250_).


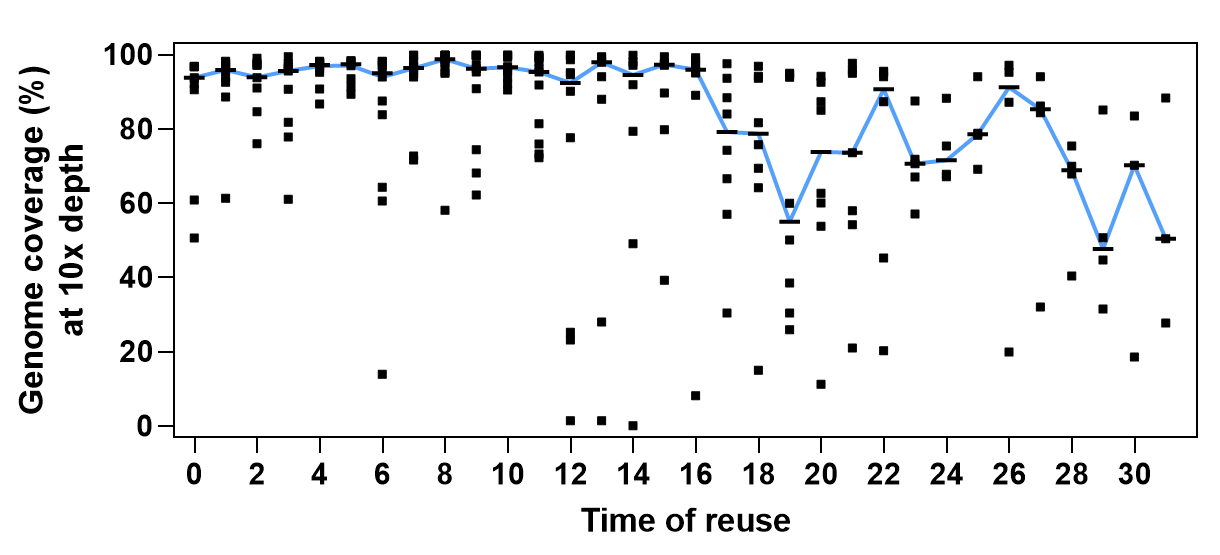


**Figure S2**. SARS-CoV-2 genome coverage of tangential-flow ultrafiltration samples over the times of membrane reuse.

**Figure S3**. Alpha diversity indices of SARS-CoV-2 communities at the lineage level recovered by Nanotrap® microbiome particles (Nano) and tangential-flow ultrafiltration (TFF).



**Figure S4.** Heat map of read depths of SARS-CoV-2 amplicon sequencing from wastewater samples. The average depth was calculated for every 500-nucleotide region across the whole genome. Each column in the plot represents one wastewater sample.


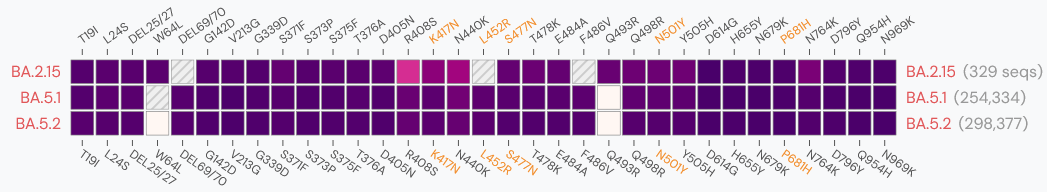


**Figure S5**. S gene mutation comparison for SARS-CoV-2 lineages BA.2.15, BA.5.1, and BA.5.2. The results were obtained from [outbreak.info](https://outbreak.info/) lineage comparison function (Gangavarapu et al., 2023).

**Figure S6**. Total RNA (ng/µL) extracted from wastewater samples concentrated by tangential-flow ultrafiltration of 125 mL wastewater (TFF_125_), tangential-flow ultrafiltration of 250 mL wastewater (TFF_250_), and Nanotrap® microbiome particles of 9.6 mL wastewater (Nano). The concentrations of RNA were quantified by Qubit Fluorometer 3.0 using BR RNA Assay kit.

**Figure S7**. Inhibitory effects for RT-qPCR analysis in nucleic acid samples prepared by tangential-flow ultrafiltration of 125 mL wastewater (TFF125) and tangential-flow ultrafiltration of 250 mL wastewater (TFF250). Nucleic acids were not diluted (no-dilution), or diluted 5-fold (5×), 10-fold (10×) with nuclease-free water.

Table S1 to Table S6 were provided in a separate XLSX file.

**Table S1.** Dunn’s multiple comparison tests of median SARS-CoV-2 genome coverages from wastewater collected in small- (<10 MGD, n=47), medium- (10-100 MGD, n=64), and large-scale (>100 MGD, n=26) wastewater treatment facilities.

**Table S2.** Pair comparison SARS-CoV-2 lineages and variant groups sequenced from wastewater samples (n = 43) processed using Nanotrap® (Nano) and tangential-flow ultrafiltration (TFF) methods

**Table S3.** Detailed information of wastewatwer samples (n=12) processed using Nanotrap® (Nano) and tangential-flow ultrafiltration (TFF) and read classifications based on whole-transcriptome shotgun sequencing.

**Table S4.** Read counts and fractions (reads/total classified viral reads) of RNA virus families detected in wastewater samples processed by Nanotrap® (Nano) and tangential-flow ultrafiltration (TFF).

**Table S5.** Wilcoxon matched-pairs signed rank tests of median read fractions of RNA virus families sequenced from the Nanotrap® and TFF samples. Virus families with p<0.05 were highlighted in grey, and the ratio of median read fractions between the two methods was calculated.

**Table S6.** Read counts of top genera of Prokarya and Eukarya in wastewater samples.

References:

Gangavarapu, K., Latif, A.A., Mullen, J.L., Alkuzweny, M., Hufbauer, E., Tsueng, G., Haag, E., Zeller, M., Aceves, C.M., Zaiets, K., Cano, M., Zhou, X., Qian, Z., Sattler, R., Matteson, N.L., Levy, J.I., Lee, R.T.C., Freitas, L., Maurer-Stroh, S., Core, G., Curation, T., Suchard, M.A., Wu, C., Su, A.I., Andersen, K.G. and Hughes, L.D. 2023. Outbreak.info genomic reports: scalable and dynamic surveillance of SARS-CoV-2 variants and mutations. Nat Methods 20(4), 512-522.
